# Supplementary material for: Exponential increase in mortality with age is a generic property of a simple model system of damage accumulation and death
Source: PLoS One. 2020 Jun 4;15(6):e0233384. doi: 10.1371/journal.pone.0233384 (PMC7272078; doi:10.1371/journal.pone.0233384)
Supplement: S1 Appendix — (PDF) [file pone.0233384.s001.pdf]

Appendix

Comparison with numerical simulations

To derive the approximations in Eq 6 and Eq 9, two assumptions were made. First, the expressions in the exponentials were approximated with linear functions of age, and, second, the rate of change of μ was assumed to be small in comparison to the time it takes for Q to reach the stationary distribution. Moreover, the result in Eq 9, is derived

Fig A1. Hazard rates estimated from numerical simulations and compared to predictions based on Eq 6. **A**: Hazard rates (points and 95% confidence intervals) for three different values of the threshold, θ; red: $\theta = 120$, green: $\theta = 100$, and blue: $\theta = 80$. The values of the other parameters were $\lambda = 500$, $\mu_0 = 550$, $\beta = 0.485/365$. Solid lines show the predictions based on Eq 6. **B**: Hazard rates (points and 95% confidence intervals) for three different values of the slope, β; red: $\beta = 0.52/365$, green: $\beta = 0.485/365$, and blue: $\beta = 0.45/365$. The values of the other parameters were $\lambda = 500$, $\mu_0 = 550$, $\theta = 100$. Solid lines show the predictions based on Eq 6.

through a diffusion approximation which is only valid for ρ close to 1 (the so called heavy traffic approximation). To demonstrate the validity of the approximations, these analytical results are, in this section, compared to data from numerical simulations of a number of model systems.

Numerical simulations of damage accumulation is straightforward (see for example [1, p.288-89]), but can become time-consuming when λ is large. In each case below, for each combination of parameter values, a large number of model instances were simulated and the time points of death were saved and used later for comparison with the theoretical predictions. The models were run in a regime where they generate mortality data similar to that observed in human populations. The smallest time unit was one day, and parameters were chosen so that the expected life length of the models was similar to that observed in humans (c:a 75 years). This choice was made for illustrative purposes, and the match between simulations and analytical approximations holds more generally. Hazard rates for the simulated data were estimated by approximating the hazard in age-bin $(a, a + 1/2]$ by the number of deaths in this interval, divided by the total amount of time spent in the interval.

Note that a necessary condition for an existence of a stationary distribution of Q is that $\rho \leq 1$. However, when the models are simulated numerically ρ can be allowed to become larger than one (this will was used in Section 4 in the main text).

The case of an M/M/1 queue

Here the baseline model was defined by the following parameters: $\lambda = 500$, $\mu_0 = 550$, $\beta = 0.485/365$, all in units of per day, and $\theta = 100$. The two latter parameters were varied, and values are given in the figure legend. For each set of parameter values, 400000 realizations were simulated. Figure A1 shows the results from these simulations. It is clear that the approximation of Eq 6 gives a very accurate account of how the hazard rates depend on age for these parameter values.

The general case

To show that Eqs. 7 and 9 are good approximations in the case when damage accumulation is not modeled as an M/M/1 queue, two types of systems were simulated. In the first case, the inter-interval distributions of damage and repair were assumed to have uniform distributions. In the second case, log-normal distributions, with unit variance, were used. Note that the coefficients of variation are independent of age in both cases, and are smaller than 1 in the first case and large than 1 in the second.

Figure A2 shows that the approximations can capture the relation between age and hazard rates very well also in these cases. The linear approximation (i.e., Eq 9) slightly underestimates the hazards when the hazard rates are below 0.01, but for larger hazard rates the errors are small. Note, however, that the approximations based on Eq 7 are derived under the assumption that damage accumulation can be approximated by a

Fig A2. Hazard rates estimated from numerical simulations and compared to predictions based on Eq 7 and Eq 9. **A**: Hazard rates (points and confidence intervals) estimated from simulations of a system where damage occurred with an average rate of 1500 per day, according to a uniform distribution on $(0, 2/1500)$. The distribution of repair times was also uniform with a age-dependent rate according to $\mu = 1550 - \beta a$. The values of the other parameters were $\beta = 0.485/365$, and $\theta = 102$. Solid lines show the predictions based on Eq 7 (orange), and Eq 9 (blue). **B**: Estimated hazard rates (points and 95% confidence intervals) from a system where damage occurred with an average rate of 550 per day, according to a log-normal distribution with unit variance. The distribution of repair times was also log-normal with an age-dependent rate according to $\mu = 550 - \beta a$. The values of the other parameters were $\beta = 0.485/365$, and $\theta = 172$.

Fig A3. Effects of using a soft threshold in the M/M/1 model. **A**: The four sigmoids corresponding to $s = 1, 2, 5,$ and 10. **B**: corresponding hazard rates. Values obtained by numerical integration of Eq 10. Black line show the hazards corresponding to a hard threshold (i.e., $s = 0$).

continuous variable, and might consequently not work well with a too low threshold (see e.g., [2, Ch.7-8] for more on this approximation).

A soft threshold

In the main text, the rate of dying was either zero, when $Q(t) < \theta$ or constant for $Q(t) \geq \theta$. Such a hard threshold might not be very realistic and in this section it is shown that a soft threshold gives similar results. That is, assume that amount of accumulated damage is related to hazard rate through a sigmoid function

$$\pi(a) = \frac{1}{1 + \exp(\frac{\theta - Q(a)}{s})}. \tag{10}$$

Here θ determines the location of the sigmoid, and acts like a threshold parameter, as before, and s is a scale parameter determining the slope of the sigmoid. When $s \to 0$, the sigmoid approaches a unit step function, so for small s, Eq 10 will be similar to Eq 3, i.e., the hard threshold. To investigate how Eq 10 depends on s more generally, the expression was evaluated numerically for different values of ρ. Numerical integration of Eq 10 was done by Monte Carlo integration, using the fact the the stationary distribution of $Q(t)$, in the M/M/1 case, is a geometric distribution.

Figure A3 shows that for $s \leq 2$, the hazard rates are indistinguishable from the hard threshold case. When $s = 5$ the hazard rates for $\rho < 0.95$ are slightly above those of the hard threshold, but for $\rho > 0.95$ the model has an almost exponential dependence between age (ρ) and hazard rates. For $s = 10$, however, the relationship is no longer exponential. This demonstrates that the exponential dependence between age and mortality is not crucially dependent on using a hard threshold. However, the relation between queue length and hazard rate must be nonlinear, in particular the case $s = 10$ shows that a linear relation between queue length and hazard rate would not lead to an exponential curve in this model.

References

1. Kroese DP, Taimre T, Botev ZI. Handbook of Monte Carlo methods. Hoboken, New Jersey: John Wiley & Sons; 2011.

2. Newell G. Applications of Queueing Theory. 2nd ed. London: Chapman & Hall; 1982.
