# Supplementary material for: Exponential increase in mortality with age is a generic property of a simple model system of damage accumulation and death
Source: PLoS One. 2020 Jun 4;15(6):e0233384. doi: 10.1371/journal.pone.0233384 (PMC7272078; doi:10.1371/journal.pone.0233384)
Supplement: S1 File — (ZIP) [file pone.0233384.s002.zip › figure6.pdf]

# Figure 6 in 'Exponential increase in mortality with age is a generic property of a simple model system of damage accumulation and death'

## Introduction

This R markdown file contains the code needed to read data and reproduce Figure 6 in the paper.

### Read the data and load required packages

```
require(data.table)
require(epitools) ## need the pois.exact from this package
require(nleqslv) ## need nleqslv from this package
## for plotting
require(ggplot2)
require(gridExtra)
require(latex2exp)
## load the data
##dead <- read.table(gzfile("dataForFigure6.csv.gz"),header=TRUE,sep=",")
dead <- read.table("dataForFigure6.csv",header=TRUE,sep=",")
dead <- data.table(dead)
dead$censor <- 1
```

### Estimate hazards rates

```
## the two birth years
yrs <- c(1885,1905)
## sex is coded as K (Swedish for kvinnor = women), and M for men
slist <- c("K","M")
## estimate piecewise hazards by assuming that those that die during a year
## die in the middle of the year
dead$age <- floor(dead$T)
mm <- dead[,.(ndead=.N),by=c("age","Sex","BirthYear")]
mm <- mm[order(BirthYear,Sex,age)]
mm$N <- 0
mm$fup <- 0
mm$haz <- 0
for (i in 1:length(yrs)){
    for (s in 1:2){
        indx <- mm[,BirthYear==yrs[i] & Sex==slist[s]]
        tmp <- mm[indx]
        cs <- cumsum(tmp$ndead)
        Ntot <- cs[length(cs)]
        mm$N[indx] <- c(Ntot,Ntot-cs[1:(length(cs)-1)])
        mm$fup[indx] <- mm$N[indx]-0.5*mm$ndead[indx]
        mm$haz[indx] <- mm$ndead[indx]/mm$fup[indx]
```

```
    }
}
```

### Create data frames suitable for plotting and compute the linear fits to the log hazards

```
df <- data.frame()
cf <- data.frame()
lfit <- data.frame()
sliste <- c("women","men")
## we need to save the me data sets for least squares later on
cut <- seq(0,105,by=1)
rkn <- 1
for (i in 1:length(yrs)){
    for (s in 1:2){
        tmp <- mm[BirthYear==yrs[i] & Sex==slist[s] & age <106]
        poapp <- pois.exact(tmp$ndead,tmp$fup)
        df <- rbind(df,data.frame(age=tmp$age,hazard=poapp$rate,upper=poapp$upper,
                                  lower=poapp$lower,year=yrs[i],sex=sliste[s]))
        ## produce linear fits to the log hazard in the range 55 to 100
        fit <- lm(log(haz+1.0e-4)~age,data=tmp,subset=age<100 & age > 54)
        cf <- rbind(cf,data.frame(c1=fit$coefficients[1],c2=fit$coefficients[2],
                                  year=yrs[i],sex=sliste[s]))
        ## linear predictions
        age <- 55:105
        dum <- fit$coefficients[1]+fit$coefficients[2]*age
        lfit <- rbind(lfit,data.frame(age=age,hazard=dum,year=yrs[i],sex=sliste[s]))
        rkn <- rkn+1
    }
}
```

### Calculate the model parameters given these linear fits

```
beta <- 0.485
lambda <- 500
mu0 <- 550

myfun <- function(x){
    ## x[1] is lambda, x[2] is k
    y <- numeric(2)
    y[1] <- x[2]*log(x[1]/(mu0-beta*55))- (cf$c1[selset]+cf$c2[selset]*55)
    y[2] <- x[2]*log(x[1]/(mu0-beta*90))- (cf$c1[selset]+cf$c2[selset]*90)
    return(y)
}

init <- c(500,100)
cf$lambda <- 0
cf$thresh <- 0
for (i in 1:4){
    selset <- i
```

```
    fit <- nleqslv(init,myfun)
    cf$lambda[i] <- fit$x[1]
    cf$thresh[i] <- fit$x[2]
}
```

## Plot the data using ggplot

```
## sizes of plotting parameters
psize=0.9
lsize=0.5
lsize2=1
## text size for axis labels
atextsize <- 14
## text size for axis title
textsize <- 14

## first plot showing the mortality rates for the whole age range
pl1 <- ggplot(data=df,aes(x=age,y=log(hazard),color=as.factor(year)))+facet_wrap(.~sex)+
    geom_point(size=psize) + geom_line(linetype="dashed")+theme_bw()+
    theme(strip.text.x = element_text(size=textsize), panel.grid.minor=element_blank(),
          legend.title=element_blank(),legend.position=c(0.12,0.8),
          axis.text=element_text(size=atextsize),
          axis.title=element_text(size=textsize),legend.text=element_text(size=textsize),)+
    ylab(label=TeX("log hazard rate"))+xlab(label=TeX(""))+
    geom_ribbon(aes(ymax=log(upper),ymin=log(lower),fill=as.factor(year)),alpha=0.3)

## and them a plot only over age > 54, with the model fits superimposed
dum <- df[df$age >54,]
## plot only every second year to improve visibility
dum <- dum[(dum$year %% 2)==1,]
## make a data frame with the predictions from the model
mfit <- data.frame()
rkn <- 1
for (i in 1:length(yrs)){
    for (j in 1:length(sliste)){
        mfit <- rbind(mfit,data.frame(age=age,
                                      hazard=cf$thresh[rkn]*log(cf$lambda[rkn]/(mu0-beta*age)),
                                      year=yrs[i],sex=sliste[j]))
        rkn <- rkn+1
    }
}

pl2 <- ggplot(data=dum,aes(x=age,y=log(hazard),color=as.factor(year)))+geom_point(size=psize) +
    geom_line(data=lfit,aes(x=age,y=hazard,color=factor(year)))+facet_wrap(.~sex) +
    geom_line(data=mfit,aes(x=age,y=hazard,color=factor(year)),size=0.5)+theme_bw()+
    theme(strip.text.x = element_text(size=textsize),panel.grid.minor=element_blank(),
          legend.title=element_blank(),legend.position=c(0.12,0.8),
          axis.text=element_text(size=atextsize),axis.title=element_text(size=textsize),
          legend.text=element_text(size=textsize),)+
    ylab(label=TeX("log hazard rate"))+xlab(label=TeX("age (years)"))

pg <- grid.arrange(pl1,pl2,nrow=2)
```

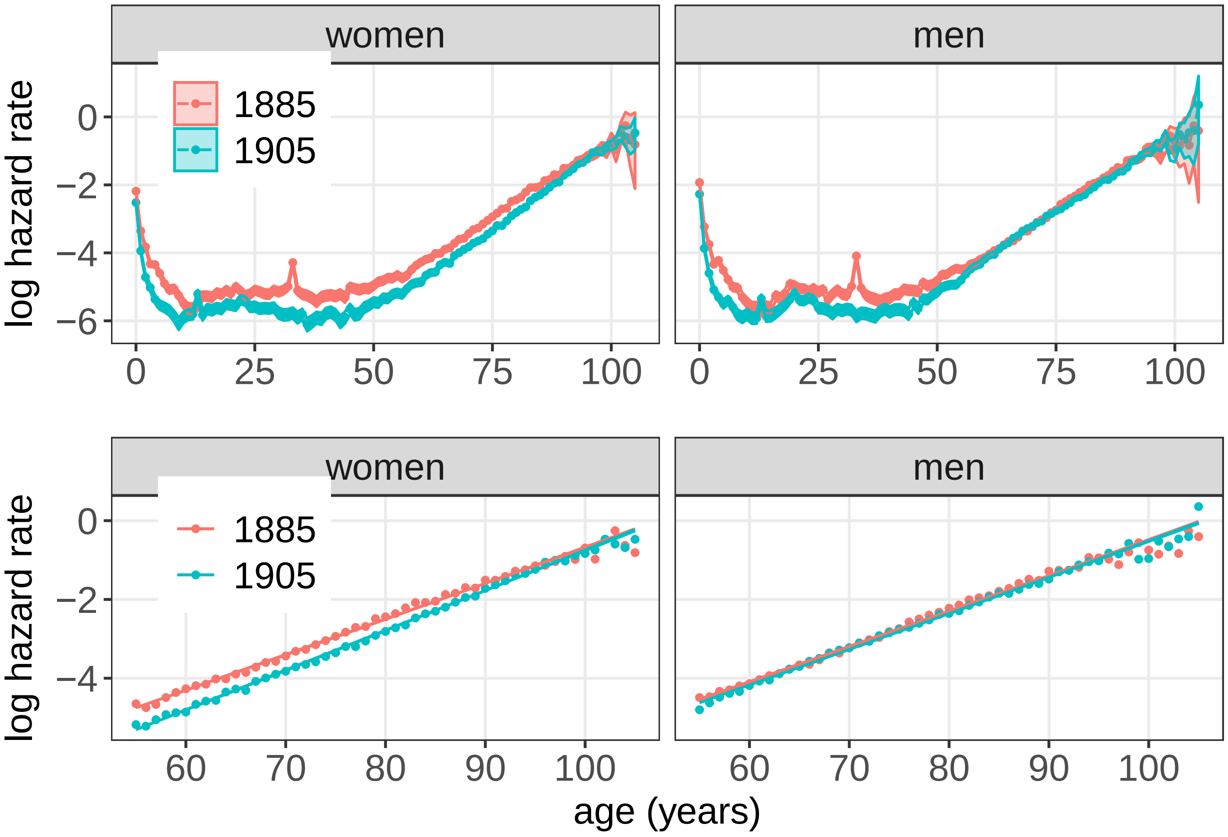
women
men
1885
1905
log hazard rate
0
−2
−4
−6
0
25
50
75
100
women
men
1885
1905
log hazard rate
0
−2
−4
60
70
80
90
100
age (years)
