# Supplementary material for: Exponential increase in mortality with age is a generic property of a simple model system of damage accumulation and death
Source: PLoS One. 2020 Jun 4;15(6):e0233384. doi: 10.1371/journal.pone.0233384 (PMC7272078; doi:10.1371/journal.pone.0233384)
Supplement: Attachment — Submitted filename: response_to_reviewers.pdf [file pone.0233384.s004.pdf]

I would like to thank the reviewers very much for their helpful comments and suggestions. I feel a bit embarrassed about the shortcomings of the manuscript, in particular that I had missed the very relevant references suggested by reviewer one, and that I was not able to better connect my work to the the "big picture", as reviewer two puts it. I have modified the Discussion in an attempt to comply with these comments and suggestions. Due to the new text inserted, I had to change the structure of the Discussion slightly. For example, there is a new section 7.2, and previous section 7.2 is now 7.3. I apologize if this makes it harder to compare the new version to the old one. However, all the new text is highlighted in red to make this comparison easier.

Below I reply to the comments and indicate where in the text I have made changed to accommodate these. Hopefully the revised version has less shortcomings than the first version had.

Reviewer #1

Critical references missing
Thank you very much for pointing this out. I had in fact read some of the earlier papers from Mitnitski & Rockwood and co-workers on the frailty index, but I had completely missed the new papers mentioned. I was thrilled to read about the model developed in these (new) papers as it is so highly related to the one I use. I apologize for not having discussed this work in the previous version of the manuscript. (BTW this is really an excellent example of when peer review works as intended). In the current version I now discuss both the frailty index, and how it could be related to damage accumulation in "my" model, and I also point out the parallels to model put forth in Mitnitski et al 2013. This is added to section 7.3. I hope I have done justice to this important work. A more in-depth comparison between the two approaches is perhaps better left for a future communication.

Another useful reference
Thank you for drawing my attention to this great reference that I unfortunately failed to include in the manuscript. It is indeed very related and I now refer to it in the appropriate place the Introduction.

How essential is the introduction of the threshold?
This is an interesting point. To get exponentially increasing hazard rates from the model, the relation between the accumulated damage and hazard rate must in fact be nonlinear. However, it does not need to be sigmoidal (the function used in the appendix). However, with a linear function (as the reviewer suggests) the hazard rates will not increase exponentially. Already with a less steep sigmoidal function (s=10) there is quite some deviations from an exponential increase (see figure B1). The nonlinearity is needed since the model is considering the momentarily accumulated damage (queue length) as the state variable determining the probability of dying. Even at young "age" there is a non-zero probability that the damage takes on large values (due to the randomness of the sample paths of the queue), and with a linear function connecting queue length and hazard, the mortality at young ages would be too high (i.e. not exponential). That failure (death) results from a threshold-passing phenomena is perhaps not too unrealistic. In fact, many systems, both biological and physical, show a non-linear relation between some variable indicating the "stress" of the system and probability of failure. I have now added a sentence in the Appendix where the inadequacy of a linear relation between queue length and mortality is made explicit.

Quasi-stationary distribution
This is a great suggestion, thank you. I will definitely look into this for future work. As the reviewer points out, I did made sure that I the models were run in regimes where the change in parameters was "slow enough" to not invalidate the stationary theory. Perhaps quasi-stationary distribution would be a way to investigate the behavior of the model as the traffic intensity is passing 1. Relying

on numerical simulations is fine but takes considerable time, and if there is some applicable theoretical approach to this regime it would be great.

Reviewer #2

I would like to thank this reviewer for the thoughtful comments on the role of modeling and for encouraging me to aim at "the big picture". I see that the manuscript might come across just as "an effort to generate equations to describe a biological phenomenon". I have now added two conjectures on how the model relates to mortality data, and I have also tried to better emphasize the generic aspects of the model. I hope that this will make the work more easy to appreciate also for non-mathematicians.

The reviewer also had two more concrete suggestions and I will discuss these below.

Place this mathematical model within the context of the evolutionary theory of senescence.
Thank you for this interesting suggesting. Unfortunately I am no expert on the evolutionary theory of senescence, but I have now added a new section to the Discussion (7.2) where I mention which variables in the model evolution could "work on". Moreover, one of the conjectures introduced in this section is about to how the model relates to inheritance of longevity. I am afraid that a more thorough attempt to connect the model with evolutionary theories of senescence will have to be made in a separate contribution.

Speculate on how these systems might be artificially manipulated by aging science.
This is a difficult one, I have to admit. At this stage the model is abstract, thus the parameters do not necessarily correspond to a unique identified biological mechanism. Still, it is clear that there is one crucial parameter in the model that inevitably leads to death, and that is the rate of decay of the repair capacity. This parameter, in a sense, represent aging. If this rate could be slowed down, the effect on longevity would be substantial. However, without tying this parameter to tentative biological mechanisms, this statement is circular: if we slow aging we live longer. I hope that the new section 7.2 in the Discussion, where I discuss how the model parameters are related to mortality data from aging individuals, provides sufficient speculation on this matter. Note in particular that the second conjecture I put forth relates to how inheritable differences in longevity could be accounted for by the model. This could consequently give guidance to potential outcomes of manipulations.
